# Post-acute Sequelae of SARS-CoV-2 Infection: Do Indians Fare Better?

**DOI:** 10.7759/cureus.32159

**Published:** 2022-12-03

**Authors:** Suja Lakshmanan, Bhargavi MV, Anbukrithiga Dharmalingam, Emmanuel Bhaskar, Archa A Anil, N. Senthil

**Affiliations:** 1 Internal Medicine/General Medicine, Sri Ramachandra Institute of Higher Education and Research, Chennai, IND; 2 General Medicine, Sri Ramachandra Institute of Higher Education and Research, Chennai, IND; 3 Internal Medicine, Sri Ramachandra Institute of Higher Education and Research, Chennai, IND

**Keywords:** post-covid sequelae, quality of life post-covid, post-covid in indian population, covid-19, post-covid-19, quality of life, covid long haul syndrome, persistent symptoms in covid-19

## Abstract

Introduction: The uplift of SARS-CoV-2 infection has necessitated the understanding of long-term consequences in the affected population. This study was driven by a lack of Indian studies to estimate the torment of post-coronavirus disease 2019 (COVID-19) symptoms and the quality of life.

Methods: This prospective observational study was conducted in a tertiary care centre in South India, between August and November 2020. SARS-CoV-2 hospitalised patients were telephonically questioned regarding the persistence of symptoms along with an assessment of the quality of life using the 15D questionnaire in the first, second, and fourth weeks. Since a majority of the patients had resolution of symptoms by four weeks, the study was not extended for a longer period.

Results: The study included 476 patients whose mean length of hospital stay was 7.67 days. Of the patients, 68.7% had mild severity, 24.8% had moderate disease, and 6.5% had severe disease. About 28.4% required oxygen, 8.2% required ICU care, and 1.3% required mechanical ventilation. Myalgia (13.9%), cough (1.3%), and dyspnoea (6.1%) were the predominant persistent symptoms in the fourth-week post-discharge. All the symptoms of health-related quality of life and physical performance improved by the fourth week, which was statistically significant.

Conclusion: Our study findings are in stark contrast to the studies published from other regions of the world, which show a significant worsening of quality of life even among those with mild illness.

## Introduction

The severe acute respiratory syndrome coronavirus 2 (SARS-CoV-2) pandemic was initially identified as a cluster of pneumonia cases in Wuhan, China [1]. The most alarming aspect of this pandemic has been the high mortality rate when compared to other seasonal flu epidemics. Though the mortality is high, a greater burden is the morbidity associated with the illness. In India, which has a population of more than 130 billion, the pandemic has caused more than 43 million infections and five lakh deaths [2].

The local healthcare facilities in almost all countries have faced problems in terms of the need for large-scale testing of contacts and high-risk groups, a sudden increase in hospitalisations, the need for trained medical professionals, demand for large quantities of oxygen supply, increased demand for intensive care unit beds and ventilators, etc. Thus there was a visible strain on the finances of many countries as they had to allocate funds to create isolation and testing centres and fully equipped treatment centres even though the pandemic had a crippling effect on income generation as international trade took a hit with many countries closing their borders. From the patient’s perspective, many reported a range of problems post-illness, from minor complaints like fatigue to long-term exertional breathlessness, prolonged need for oxygen support, acute psychological stress, and acute kidney injury.

In addition to the high mortality, the illness takes a protracted course manifesting a spectrum of symptoms, which include persistent fatigue, myalgia, breathlessness, alternation in cognitive function, and sleep disturbance, resulting in a poor quality of life even when the illness is mild [3,4]. In a study by Huang et al. in Wuhan, China, about 997 patients were followed up for six months post-coronavirus disease 2019 (COVID-19) [3]. COVID-19 survivors were mainly troubled with fatigue or muscle weakness (63%), sleep difficulties (26%), and anxiety or depression (23%) [3]. A study done by Hannah E. Davis, which included 3,762 respondents from 56 countries, revealed that the most frequent symptoms reported after six months were fatigue (77.7%), post-exertional malaise (72.2%), and cognitive dysfunction (55.4%) [4]. It has been almost a while since the World Health Organization declared it a global pandemic on March 11th, 2020, but there are many uncertainties regarding the long-term sequelae of this disease. Not much data are available on the time taken for recovery and the quality of life of patients post-recovery from mild illness vs. severe illness. Despite a large population in India being affected by SARS-CoV-2, there is a lack of literature on the characteristics of long haulers in this region. This study was initiated to identify the prevalence of post-COVID-19 symptoms in a cohort of patients diagnosed and followed up at our centre during the first wave of the pandemic in India.

## Materials and methods

We prospectively studied a cohort of patients who were admitted to the COVID-19 ward, high dependency unit, or critical care unit between August and November 2020 at our centre, a tertiary care teaching hospital located in Chennai, India. All participants who were more than 18 years of age with reverse transcription-polymerase chain reaction (RT-PCR)-confirmed SARS-CoV-2 infection were included in this study. Participants in the age group of less than 18 years and patients who had negative RT-PCR for SARS-CoV-2 infection but positive serology tests were excluded. Patients discharged without fulfilling the discharge criteria, including against medical advice, were also excluded from this study. The sampling method used was consecutive sampling. The sample size of the study was not predetermined since it was a period-dependent cohort study over four months. About 492 patients who consented to a follow-up of their symptoms were enrolled in this study. Around 16 participants were later excluded due to withdrawal of consent or loss of follow-up. The study was approved by the Institutional Ethics Committee of Sri Ramachandra University (IEC-NI/20/SEP/75/79) and oral telephonic consent was obtained from the study participants. Each study participant was assessed by one of the study investigators using a questionnaire, which sought information on age, gender, prior medical illness like underlying bronchial asthma and chronic obstructive pulmonary disease, and presenting symptoms. The patients' comorbid conditions like diabetes mellitus, systemic hypertension, coronary heart disease, chronic kidney diseases, and cerebrovascular accidents were noted. We then prospectively obtained information on laboratory parameters, medications received, and complications during the stay from in-patient medical records. The severity of illness was classified as mild (normal peripheral oxygen saturation), moderate (saturation: 94-90%), and severe (saturation < 90%), as defined by the Ministry of Health and Family Welfare (MOHWF)/Indian Council of Medical Research (ICMR) [5].

Chronic symptoms present prior to SARS-COV-2 infection were excluded from the follow-up assessment. Hence, only the new onset symptoms, which were likely due to COVID-19 infection, were followed up. Enrolled participants were followed up for four weeks post-discharge, and were telephonically questioned regarding the persistence or development of symptoms in the first, second, and fourth weeks. In addition to follow-up of symptoms present during hospitalisation (cough, dyspnoea, chest pain, fever, diarrhoea, giddiness, sore throat, headache, body ache, altered smell, and altered taste), symptoms which could indicate long COVID-19 were assessed. We used seven of the 15 components of the "15D quality of life questionnaire" relevant to post-acute SARS-CoV-2 infection [6]. Each of these symptoms was further assessed in a five-grade method. These were (a) difficulty in mobility, (b) comfort in breathing, (c) difficulty in sleeping, (d) eating problems, (e) usual activities, (f) depression, and (g) distress.

Categorical variables were expressed as numbers (percentage) and continuous variables were expressed as mean (standard deviation). Categorical variables were compared using the chi-square test and continuous variables with a one-way analysis of variance (ANOVA). Analysis was done using SPSS (Statistical Package for Social Sciences) version 23.0 (IBM Corp., Armonk, NY). A p-value < 0.05 was considered significant.

## Results

Around 492 patients who consented to follow-up of their symptoms were enrolled from August 2020 to November 2020. About 16 patients were later excluded due to withdrawal of consent or loss of follow-up. The response rate of the study was 96.3%. The total number of patients who were included in this study was 476. The mean age of the studied patients was 48.84 years (range: 19-85 years), and most were males (63.4%). The mean length of hospital stay was 7.67 days. Most participants had mild severity (n = 327, 68.7%), followed by moderate (n = 118, 24.8%) and severe disease (n = 31, 6.5%). About 28.4% (n = 135) required oxygen, 8.2% (n = 39) required ICU care, and 1.3% (n = 6) required mechanical ventilation. Table 1 explains the characteristics of the study participants.

**Table 1:** Baseline characteristics of the study participants

Clinical parameter	Mild	Moderate	Severe	P-value
	n = 327	n = 118	n = 31	
Age in years (mean ± SD)	45.9 ± 14.9	55.9 ± 13.9	53.5 ± 11.8	0.003
Gender, No. (%)				<0.001
Male	186 (61.6)	89 (29.5)	27 (8.9)	
Female	141 (81)	29 (16.7)	4 (2.3)	
Comorbidities, No. (%)				-
Diabetes mellitus	107 (32.7)	66 (55.9)	20 (64.51)	
Hypertension	81 (24.7)	51 (43.2)	13 (41.93)	
Coronary artery disease	12 (3.6)	12 (10.16)	0	
Chronic kidney disease	2 (0.06)	9 (7.62)	1 (33.33)	
Cerebrovascular accident	2 (0.06)	2 (1.69)	0	
Chronic airway disease	7 (2.14)	8 (6.77)	1 (33.33)	
Liver disease	0	1 (0.84)	1 (33.33)	
Others (dyslipidemia, hypothyroidism, pulmonary tuberculosis, obesity, and seizure disorders)	37 (11.3)	22 (18.64)	4 (12.90)	
Presenting symptoms, No. (%)				
Fever	288 (88.07)	108 (91.52)	25 (80.64)	-
Sore throat	114 (34.86)	8 (6.77)	1 (0.32)	
Dry cough	68 (20.79)	86 (72.88)	26 (83.87)	
Cough with expectoration	16 (4.89)	12 (10.16)	3 (9.67)	
Dyspnoea	23 (7.03)	79 (66.94)	25 (80.64)	
Myalgia	34 (10.39)	23 (19.49)	2 (6.45)	
Headache	10 (3.05)	2 (1.69)	2 (6.45)	
Diarrhoea	11 (3.36)	7 (5.93)	1 (3.22)	
Vomiting	3 (0.91)	1 (0.84)	-	
Altered smell	28 (8.56)	5 (4.23)	-	
Altered taste	18 (5.5)	4 (3.38)	-	
Others	5 (1.52)	3 (2.54)	1 (3.22)	
Investigations (mean/SD)				
D-dimer	0.64 (1.21)	1.10 (3.6)	1.36 (1.7)	0.038
Ferritin (ng/mL)	138.2 (143.3)	320.8 (316.1)	439.9 (334.4)	<0.001
C-reactive protein	1.8 (2.7)	6.3 (6.7)	9.6 (10.2)	<0.001

The follow-up of symptoms in the study cohort during the second and fourth weeks is shown in Table 2. The most common symptom that was persistent at the end of the second and fourth week was myalgia, followed by dyspnoea and cough. While 12.4% of the patients had myalgia as one of their presenting complaints, it increased to 56.9% at the end of the first week, which later decreased to 31.7% at the end of the second week and 13.9% at the end of four weeks. Cough decreased from 26.9% at one week to 1.3% at four weeks. Dyspnoea was the presenting symptom in about 26.7% of patients, which decreased to 6.1% at the end of the fourth week. Only 6.9% had anosmia and all but 1% recovered at four weeks. Alteration in taste was present only in 4.6% of the patients. Of the patients, 9.5% had altered taste, which reduced to 1.1% at the fourth weekend.

**Table 2:** Follow-up of symptoms and well-being after discharge

Clinical symptoms	1st week, No. (%)	2nd week, No. (%)	4th week, No. (%)
Cough	128 (26.9)	34 (7.1)	6 (1.3)
Dyspnoea	130 (27.3)	79 (16.6)	29 (6.1)
Chest pain	11 (2.3)	8 (1.7)	7 (1.5)
Fever	10 (2.1)	4 (0.8)	1 (0.2)
Diarrhoea	3 (0.6)	1 (0.2)	0
Giddiness	26 (5.5)	5 (1.1)	2 (0.4)
Sore throat	47 (9.9)	4 (0.8)	4 (0.8)
Headache	20 (4.2)	4 (0.8)	3 (0.6)
Body ache	271 (56.9)	151 (31.7)	66 (13.9)
Altered smell	56 (11.8)	19 (4)	3 (0.6)
Altered taste	45 (9.5)	16 (3.4)	5 (1.1)
Overall well-being			
1. Fatigue			
A. Able to walk normally (without difficulty) indoors, outdoors, and on stairs	233 (48.9)	317 (66.6)	400 (84)
B. Able to walk without difficulty indoors, but with slight difficulty outdoors and/or on stairs	181 (38)	146 (30.7)	74 (15.5)
C. Able to walk without help indoors (with or without an appliance), but outdoors and/or on stairs only with considerable difficulty or with help from others	59 (12.4)	12 (2.5)	2 (0.4)
D. Able to walk indoors only with help from others	3 (0.6)	1 (0.2)	0
E. Completely bed-ridden and unable to move about	0	0	0
2. Breathlessness			
A. Able to breathe normally	290 (60.9)	369 (77.5)	416 (87.4)
B. Have shortness of breath during heavy work or sports, or when walking briskly on flat ground or slightly uphill	140 (29.4)	88 (18.5)	55 (11.6)
C. Have shortness of breath when walking on flat ground at the same speed as others my age	38 (8)	18 (3.8)	5 (1.1)
D. Shortness of breath even after light activity e.g., washing or dressing myself	8 (1.7)	1 (0.2)	0
E. Breathing difficulties almost all the time, even when resting	0	0	0
3. Sleep			
A. Able to sleep normally	372 (78.2)	393 (82.6)	414 (87)
B. Have slight problems with sleeping, e.g., difficulty in falling asleep or sometimes waking at night	65 (13.7)	65 (13.7)	55 (11.6)
C. Have moderate problems with sleeping, e.g., disturbed sleep or feeling I have not slept enough	31 (6.5)	13 (2.7)	5 (1.1)
D. Have great problems with sleeping, e.g., having to use sleeping pills often or routinely or usually waking at night and/or too early in the morning	7 (1.5)	4 (0.8)	2 (0.4)
E. Suffer severe sleeplessness, e.g., sleep is almost impossible even with full use of sleeping pills or staying awake most of the night	1 (0.2)	1 (0.2)	0
4. Food intake			
A. Able to eat normally	439 (92.2)	460 (96.6)	467 (98.1)
B. Able to eat with minor difficulty (e.g., slowly, clumsily, shakily, or with special appliances)	33 (6.9)	13 (2.7)	8 (1.7)
C. Need some help from another person in eating	3 (0.6)	2 (0.4)	1 (0.2)
D. Unable to eat by self, require help for oral feeding	1 (0.2)	1 (0.2)	0
E. Fed either by tube or intravenously	0	0	0
5. Usual activities			
A. Able to perform usual activities (e.g., employment, studying, housework, and free time activities) without difficulty	233 (48.9)	355 (74.6)	429 (90.1)
B. Able to perform usual activities slightly less effectively or with minor difficulty	197 (41.4)	108 (22.7)	44 (9.2)
C. Able to perform usual activities much less effectively, with considerable difficulty, or not completely	41 (8.6)	13 (2.7)	3 (0.6)
D. Can only manage a small proportion of previously usual activities	5 (1.1)	0	0
E. Unable to manage any of the previously usual activities	0	0	0
6. Depression			
A. Do not feel sad	414 (87)	438 (92)	456 (95.8)
B. Feel slightly sad	40 (8.4)	31 (6.5)	19 (4)
C. Feel moderately sad	18 (3.8)	5 (1.1)	1 (0.2)
D. Feel very sad	4 (0.8)	2 (0.4)	0
E. Feel extremely sad	0	0	0
7. Distress			
A. Do not feel anxious, stressed, or nervous	405 (85.5)	446 (93.7)	459 (96.4)
B. Feel slightly anxious, stressed, or nervous	63 (13.2)	25 (5.3)	16 (3.4)
C. Feel moderately anxious, stressed, or nervous	6 (1.3)	5 (1.1)	1 (0.2)
D. Feel very anxious, stressed, or nervous	2 (0.4)	0	0
E. Feel extremely anxious, stressed, or nervous	0	0	0

In the next part of our study, we also observed the health-related quality of life and physical performance in our study patients post-discharge at the end of the first, second, and fourth week based on the 15D instrument of health-related quality of life (recommended by the Washington Panel) [6]. We considered seven major parameters associated with post-COVID-19 sequelae, namely, fatigue, breathlessness, sleep, food intake, physical activity, depression, and distress, and graded each parameter from A to E, as shown in Table 2.

Health-related assessment of participants showed that half of them (51%) had some form of walking difficulty requiring support. However, we excluded confounding variables like osteoarthritis and old age while assessing for difficulty in walking. None of the patients was bedridden or unable to move. At the end of the fourth week, 84% of the patients were able to walk without any difficulty. The cohort showed good recovery of breathlessness with only 1.1% having dyspnoea on walking on level ground. About one-fifth (21.9%) had sleep disturbance at week one, which decreased to 13% at week four. Close to half of the cohort (48.9%) required support for daily activity in the first week, which improved to 9.9% at four weeks. At the end of the first week, 13% were affected with mild to severe depression, which decreased to 4.2% at week four. The majority of patients were able to eat without any assistance.

## Discussion

Though most patients recover completely from the acute SARS-CoV-2 infection, a few people experience persistent symptoms, including breathlessness, cough, fatigue, sleep disturbances, psychological problems like depression or anxiety, and a poor quality of life. The "Post-COVID Syndrome" includes persistent symptoms, which could be due to residual inflammation, organ damage, prolonged hospitalisation, prolonged ventilation, social isolation, or impact on pre-existing health conditions [7]. Our study findings are in stark contrast to the studies published from other regions of the world, which showed a significant worsening of quality of life even among those with mild illness [3,4]. The mean age and male gender predominance observed in our study were comparable with prior studies with participants ranging from 48 to 70.5 years [4,8-12] and male gender prevalence of 42.9-62.9% [4,8-12]. Anosmia and ageusia observed in our study are substantially low compared to studies that have reported a frequency of 42.4-50% (anosmia) and 50% (ageusia) [8,10]. A higher proportion (31.3%) of patients with moderate to severe illness compared to 9% in other studies could explain the lower prevalence of anosmia since the symptom is more prevalent in mild illness [8-10]. We observed a high prevalence of diabetes mellitus (40.5%) and hypertension (30.5%) compared to a prevalence of 5.1-29.4% and 13-50% in prior studies [8-12].

At the end of four weeks, the symptoms which persisted were myalgia (13.9%), cough (1.3%), dyspnoea (6.1%), altered smell (0.6%), and loss of taste (1.1%). In comparison, observations from Spain and Italy showed persisting fatigue (30.5-53.1%) and dyspnoea (31.4-43.4%) [8,10,13]. An observation in France shows that at the end of 110 days, fatigue (55%) and dyspnoea (42%) were still persistent [11]. Reports on 3,762 respondents from 56 countries show that fatigue was persistent in 80% even at the end of six months, post-exertion malaise in 74%, insomnia in 40%, and dyspnoea in 38% [3].

At the end of the first-week post-discharge, the dimension of life that was most affected was mobility and activities of daily life (51.1%), followed by breathing (39.1%) and sleep (21.8%). At the end of the fourth-week post-discharge, the percentage affected decreased to 16% for mobility, 13% for sleep, 12.6% for breathlessness, and 9.9% for activities of daily life. Concerning the health-related quality of life and physical performance in our study patients post-discharge at the end of the first, second, and fourth week, all the symptoms improved at the end of the fourth-week post-discharge, which was statistically significant. Studies have shown a similar prevalence of dimensions of quality of life (mobility, self-care, pain, anxiety or depression, and usual activity) between ICU and non-ICU patients, but for a higher prevalence of pain in the ICU care group [11]. Similar lower quality of life (measured with EuroQol 5-Dimension) three months after symptom onset has been reported in the Canadian population and quality of life below the fifth percentile has been reported in the Netherlands [11,12].

Several studies with genome-wide association analysis (GWAS) reveal that several genetic factors play a vital role in the host susceptibility to SARS-CoV-2 infection influencing the disease severity and progression [14]. A genomic association is identified between angiotensin-converting enzyme 2 (ACE2) levels and susceptibility to SARS-CoV-2 infection, but no specific risk factors or genomic associations are identified for long haulers in SARS-CoV-2 infection. Also, the pathophysiology is poorly understood. Since SARS-CoV-2 infection affects multiple systems, early identification of this syndrome with a multidisciplinary approach is important in managing the affected patients post-COVID-19.

Limitations of this study include response bias or misreporting of data. Since this was a preliminary study on the Wuhan variant and lack of available studies assessing the immunological and genetic aspects in our population, we are unable to give the reasoning for the low prevalence of long COVID-19 symptoms. This study is a single-centre study and cannot be generalised to all populations. Several such studies are required in larger cohorts in more moderate to severe COVID-19 patients to analyse the post-COVID-19 sequelae and physical and mental quality of life in patients affected by SARS-CoV-2 infection.

## Conclusions

It is very well evident that SARS-CoV-2 infection is dynamic, and further studies and global data are required to understand the impact of the disease on the physical and mental well-being of the affected population. The persistent symptoms and quality of life post-COVID-19 need a long-term follow-up for a better understanding of the nature of the disease and to give better guidance for patient care.
